# Conservation of alternative splicing in sodium channels reveals evolutionary focus on release from inactivation and structural insights into gating

**DOI:** 10.1113/JP274693

**Published:** 2017-07-18

**Authors:** A. Liavas, G. Lignani, S. Schorge

**Affiliations:** ^1^ Department of Clinical and Experimental Epilepsy UCL Institute of Neurology London WC1N 3BG UK

**Keywords:** alternative splicing, evolution, patch clamp, SCN1A, sodium channel

## Abstract

**Key points:**

- Sodium channels are critical for supporting fast action potentials in neurons; even mutations which cause small changes in sodium channel activity can have devastating consequences for the function of the nervous system.
- Alternative splicing also changes the activity of sodium channels, and while it is highly conserved, it is not known whether the functional role of this splicing is also conserved.
- Our data reveal that splicing has a highly conserved impact on the availability of sodium channels during trains of rapid stimulations, and suggest that in one mammalian channel, Nav1.1 encoded by SCN1A, the increased availability of one splice variant is detrimental.
- A model reproducing the effects of splicing on channel behaviour suggests that the voltage sensor in the first domain is a rate limiting step for release of the inactivation domain, and highlights the functional specialization of channel domains.

**Abstract:**

Voltage‐gated sodium channels are critical for neuronal activity, and highly intolerant to variation. Even mutations that cause subtle changes in the activity these channels are sufficient to cause devastating inherited neurological diseases, such as epilepsy and pain. However, these channels do vary in healthy tissue. Alternative splicing modifies sodium channels, but the functional relevance and adaptive significance of this splicing remain poorly understood. Here we use a conserved alternate exon encoding part of the first domain of sodium channels to compare how splicing modifies different channels, and to ask whether the functional consequences of this splicing have been preserved in different genes. Although the splicing event is highly conserved, one splice variant has been selectively removed from Nav1.1 in multiple mammalian species, suggesting that the functional variation in Nav1.1 is less well tolerated. We show for three human channels (Nav1.1, Nav1.2 and Nav1.7) that splicing modifies the return from inactivated to deactivated states, and the differences between splice variants are occluded by antiepileptic drugs that bind to and stabilize inactivated states. A model based on structural data can replicate these changes, and indicates that splicing may exploit a distinct role of the first domain to change channel availability, and that the first domain of all three sodium channels plays a role in determining the rate at which the inactivation domain dissociates. Taken together, our data suggest that the stability of inactivated states is under tight evolutionary control, but that in Nav1.1 faster recovery from inactivation is associated with negative selection in mammals.

## Key points

- Sodium channels are critical for supporting fast action potentials in neurons; even mutations which cause small changes in sodium channel activity can have devastating consequences for the function of the nervous system.
- Alternative splicing also changes the activity of sodium channels, and while it is highly conserved, it is not known whether the functional role of this splicing is also conserved.
- Our data reveal that splicing has a highly conserved impact on the availability of sodium channels during trains of rapid stimulations, and suggest that in one mammalian channel, Nav1.1 encoded by SCN1A, the increased availability of one splice variant is detrimental.
- A model reproducing the effects of splicing on channel behaviour suggests that the voltage sensor in the first domain is a rate limiting step for release of the inactivation domain, and highlights the functional specialization of channel domains.

**Abbreviations**
Nav1: IUPHAR abbreviation for voltage dependent sodium channels
SCN: the human genome organization approved prefix for sodium channel subunit genes
S1–S6: transmembrane segments 1–3 within voltage sensing sodium channel domains
D1–D4: domains 1–4 within voltage sensing sodium channels
A exon and N exon: the two mutually exclusive 92 nucleotide long cassette exons conserved in neuronal sodium channels (the A exon occurs prior to the N exon in genomic sequences)

## Introduction

The vast majority of mammalian genes are modified by alternative splicing, but in most cases the adaptive significance of this phenomenon is unknown (Kelemen *et al*. 2013; Gerstein *et al*. 2014; Raj & Blencowe, 2015). A challenge in elucidating the functional impact of splicing on protein function is that it can be difficult to differentiate epiphenomena from changes that are biologically important. Alternative splicing that is conserved across multiple genes that diverged from a common ancestor offers an opportunity to identify changes in function that have adaptive significance (Kopelman *et al*. 2005; Su *et al*. 2006; Abascal *et al*. 2015).

**Figure 1:**
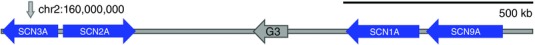
The conserved cluster of sodium channel genes on human chromosome 2 A schematic diagram indicating the relative positions of *SCN1A, SCN2A, SCN3A* and *SCN9A* in the conserved cluster on chromosome 2. In some mammalian genomes the numbering and annotation of *SCN2A*, *SCN3A* and *SCN1A* are inconsistent. In order to unambiguously identify the exon homologous to the N exon in human *SCN1A*, the identity of the sodium channel gene containing it was verified using its position relative to the other genes in the cluster, and according to the direction of transcription. The arrow labelled G3 indicates the position and orientation of the *GALNT3* gene which is conserved at this site in mammals. All aligned sequences of N exons are available upon request. [Color figure can be viewed at wileyonlinelibrary.com]

Here, we have focused on the sodium channel family where an alternative splicing event emerged early in vertebrate evolution (Copley, 2004) and has been conserved in the face of repeated duplication and specialization of the genes (Zakon, 2012). The sequences of the *SCNxA* genes (x = 1–5, 7–11) have diverged to serve different functions in the nervous system (Waxman, 2007; Catterall, 2014). In mammalian genomes, six different neuronal sodium channel genes have preserved an alternative splicing event that consistently changes one amino acid in a short extracellular linker immediately before the voltage sensor in the first domain (DIS3‐S4, Fig. 1). The two splice variants are designated ‘N’ and ‘A’ as initial studies indicated that, although both splice variants co‐exist, the ‘N’ variant may be enriched in neonatal tissue, while the ‘A’ variant tends to predominate in adult tissue (Gazina *et al*. 2010).

In spite of the conservation of this splicing among sodium channel genes, both humans (Heinzen *et al*. 2007) and rodents (Gazina *et al*. 2010) contain genetic variations in one gene, *SCN1A*, that are predicted to reduce the expression of the N exon, either because of a splice site polymorphism (human) or the presence of stop codons (rodents). There are three possibilities behind the loss of this otherwise highly conserved exon from this particular sodium channel gene. Firstly, it may be a coincidence that is only present in human and rodent *SCN1A* genes, but not seen in other species. Alternatively, the change imposed by the N exon may have a deleterious effect on the intrinsic properties of the channels encoded by *SCN1A*, which are different from the effects imposed in other sodium channels where the N exon is preserved. Finally, splicing may have the same effects in different channels, but in SCN1A those effects may have adverse consequences in neuronal networks, because SCN1A is preferentially expressed in a different subset of neurons.

To test these three possibilities, we selected three sodium channels (*SCN1A*, *SCN2A* and *SCN9A* encoding NaV1.1, 1.2 and 1.7, respectively), which predominate in different types of neurons: *SCN1A* is especially important in inhibitory neurons, *SCN2A* in excitatory neurons, and *SCN9A* in pain‐sensing neurons of the peripheral nervous system. These genes are also associated with distinct neurological diseases in humans, including different genetic epilepsies (*SCN1A* and *SCN2A*) and pain disorders (*SCN9A*) (Waxman, 2007; Catterall, 2014).

Sodium channel genes are already known to be highly intolerant to change. The recently published ExAC database indicates that *SCN1A* has a Z score of 5.61 for missense changes, indicating it is more than 5 standard deviations below the average number of expected missense changes for a gene of this size (Lek *et al*. 2016). SCN2A is even more extreme, with a Z score of 6.58, while SCN9A, in contrast is not tightly constrained with a Z score of −0.69. For *SCN2A*, the majority of mutations associated with epilepsies seem to introduce gain of function changes to the channels (Scalmani *et al*. 2006; Liao *et al*. 2010) with few truncations reported. For *SCN1A* the story may be more mixed. In addition to the epilepsies associated with haploinsufficiency or missense mutations, including some with relatively subtle effects (Catterall, 2014), severe migraine may be caused by gain of function missense mutations (Cestèle *et al*. 2013). Thus *SCN1A* may be unusual in sodium channels because of the association of both subtle increases and decreases in function to disease.

We report that loss of the N exon in *SCN1A* is not confined to humans and rodents, but represents a broad trend in mammals. By directly comparing N and A variants of all three sodium channels, we reveal that alternative splicing has a conserved biophysical function in this gene family. We show that the N exon robustly increases channel availability after brief depolarizations compared to the A exon, which inserts a negatively charged aspartate residue in the extracellular S3‐S4 linker. The N splice variants of all three channels are therefore more able to respond to high frequency stimulations. The conserved functional impact of splicing suggests that the loss of exon N in *SCN1A* is due to a specific constraint on the role of these channels, rather than a different impact of splicing on channel function. In contrast, in *SCN2A*, the splicing is highly conserved, in spite of the intolerance of this gene to variation, suggesting that the change imposed by splicing in *SCN2A* is not detrimental.

## Methods

#### Consensus sequences used for Ka/Ks analysis

Synthetic ‘1N variant’(vr) and ‘1N ancestral’(an) sequences used for comparison of variation accrual in 23 different genomes. For SCN2A all three changes were silent, and did not change the predicted sequence, and for SCN9A both changes observed were also silent. SCN1A contained a mix of silent and missense changes. Exons in SCN1A which imposed frameshifts or stop codons were not used for this comparison. Only exons predicted to produce full length proteins were used. Thus this analysis represents a conservative estimation of the amount of non‐synonymous (Ka) changes.

SCN1A

An

GTATGTAACAGAATTTGTAAACCTAGGCAATTTTTCAGCTCTTCGCACTTTCAGAGTCTTGAGAGCTTTGAAAACTATTTCTGTAATTCCAG

Vr

GGTTTTTAACGAATTTGTAAGATTAGGCAGATTTTCAACTGTCCAAATTTACAGAGTCTTGAGAATTTTGGAACCTATTTCGATAGCTCCAG

SCN2A

An

GTATGTAACAGAATTTGTAAACCTAGGCAATGTTTCAGCTCTTCGAACTTTCAGAGTCTTGAGAGCTTTGAAAACTATTTCTGTAATTCCAG

Vr

GTATGTAACAGAATTTGTAAACCTAGGCAATGTTTCAGCTCTTCGCACTTTCAGAGTATTGAGAGCTTTGAAAACTATTTCTGTAATCCCAG

SCN9A

An

GTATTTAACAGAATTTGTAAACCTAGGCAATGTTTCAGCTCTTCGAACTTTCAGAGTATTGAGAGCTTTGAAAACTATTTCTGTAATCCCAG

Vr

GTATTTAACAGAATTTGTAAACCTAGGCAATGTTTCAGCTCTTCGAACTTTCAGAGTGTTGAGAGCTTTGAAAACTATTTCTGTAATTCCAG

#### DNA constructs and cloning

Human Nav1.1 cDNAs in the pcDM8 vector were transformed into TOP10/P3 cells. Nav1.2 and Nav1.7 cDNAs were in pcDNA3 vectors and transformed into Stbl3 cells. Spontaneous mutagenesis of sodium channel gene constructs when propagated in bacterial cultures is not uncommon (Mantegazza *et al*. 2005). To minimize this, incubation temperature of bacterial cultures was kept below 30°C, and confluence was lower than an OD600 nm value of 0.35. Close monitoring of cell growth together with lower temperature conditions and use of a low‐copy plasmid reduced mutation rate and DNA nicking on sodium channel gene plasmids. Mutation‐free cloning was verified for all constructs by DNA sequencing.

#### Transfection of HEK‐293T cells

Heterologous expression of splice variants of Nav1.1, Nav1.2 and Nav1.7 was performed in HEK‐293T cells (Chatelier *et al*. 2008; Fletcher *et al*. 2011; Thompson *et al*. 2011). Cells were transiently co‐transfected with 0.5 μg of the sodium channel plasmid and a reporter EGFP‐containing plasmid in a 3: 1 molar ratio using Lipofectamine 2000 (Invitrogen), according to the manufacturer's instructions. Recordings were performed 36–72 h after transfection. Non‐transfected cells showed no detectable sodium currents.

#### Whole cell patch clamp recordings in HEK cells

For all recordings, the extracellular (bath) solution was: (in mm): 135 NaCl, 10 HEPES, 2 MgCl_2_, 1.8 CaCl_2_, 4 KCl, pH 7.35). Intracellular solution was (in mm): 145 CsCl, 5 NaCl, 10 HEPES, 10 EGTA, pH 7.35). Liquid junction potential was calculated to be −4.4 mV and was not corrected. Cells were patched and all recordings were carried out at physiological temperature (37°C +/− 2°C). Errors due to series resistance were minimized by discarding cells with high series resistance (>3 MΩ), large currents (>5 nA) or capacitance (>35 pF), but series resistance was not compensated as this introduced unacceptable artefacts in these conditions. Peak currents <600 pA were discarded from the analysis to avoid distortion by endogenous currents. Data were leak‐subtracted using a P/4 protocol. HEK cells were held at −80 mV and allowed 4 seconds recovery between depolarizing sweeps. To control for variation between transfections, data were collected from at least three different transfections.

#### Voltage dependence of steady‐state activation/inactivation

Starting from a holding potential of −80 mV, cells were stepped to a range of potentials (−120 mV to +60 mV in 10 mV increments for 300 ms). Normalized conductance values against membrane potential were fitted in Origin 8.0 with a Boltzmann function, G/Gmax = 1/(1 + exp ((V50 − V)/k)) to determine the half‐maximal channel activation voltage (V50) and the slope factor (k). G/Gmax is the normalized conductance value at any given potential (V).

The voltage dependence of fast inactivation was determined by measuring the peak sodium current during a 30 ms test pulse to −10 mV, after a 300 ms prepulse between −120 to +40 mV with 10 mV increments, starting from a holding potential of −80 mV. Peak currents from each step were normalized to the maximum response for each cell. Data were fit with a Boltzmann function, INa = (A + (B − A))/(1 + exp ((V50 − V)/k)), where INa is the fraction of sodium current that is available at any given membrane potential (V).

#### Stability of inactivation

Recovery from inactivation was analysed using a two‐pulse protocol with the first pulse (P1, to −10 mV) lasting 100 ms, and varying time intervals at −80 mV recovery before a second test pulse (P2, to −10 mV) to measure channel availability. To determine the effects of length of P1 on availability, the recovery interval at −80 mV was fixed at 2 ms, and the length of P1 allowed to vary.

The recovery time course for all three channels was fit by two exponentials [Y_0_ + A_F_^*^(1 − exp(−t/τ_F_)) + A_S_^*^(1 − exp(−t/τ_S_))]. Availability after variable inactivating P1 lengths was fit with a first order exponential decay (Y_0_ + A^*^exp(−t/τ)).

### Statistics

Results are shown as mean ± s.e.m. Data were tested for normality using the Shapiro‐Wilk normality test. Normally distributed two sample groups were compared by Student's unpaired two‐tailed *t* test, at a significance level of *P* < 0.05. Sample groups without a normal distribution were compared using Fisher's exact test (two‐tailed). For comparisons of more than two sample groups, ANOVA was used, followed by either the Bonferroni's or the Dunnett's test. Fitting used the Levenberg‐Marquardt algorithm to minimize the χ2 value, with the amplitudes of rates constrained to give positive values. Statistical analysis was carried out using the Prism software (GraphPad Software, Inc.), Origin (OriginLab) or SPSS (IBM). All modelling was carried out using IonChannelLab (Santiago‐Castillo *et al*. 2010). All modifications to rates were constrained to preserve microscopic reversibility in the model.

## Results

### Molecular evolution suggests maladaptive role of the N exon of NaV1.1 in mammals

**Figure 2:**
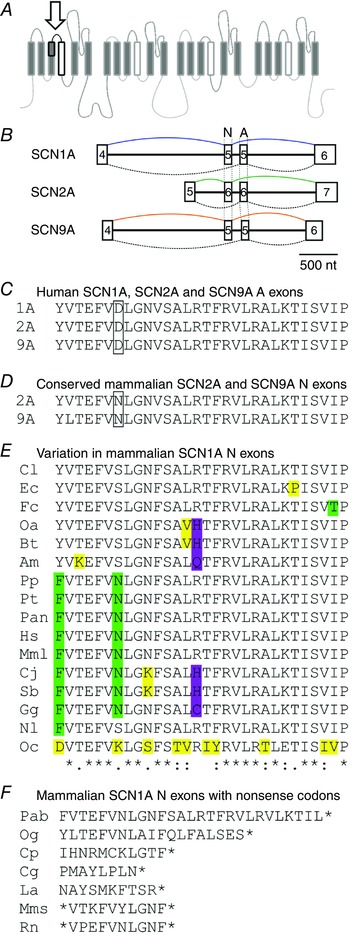
The N exon has been destroyed in multiple mammalian copies of SCN1A *A*, cartoon indicating the site of the single amino acid changed in the short extracellular linker between the third and fourth transmembrane segments in domain 1 (D1 S3–S4, arrow). The overall length of these channels is typically >2000 amino acids. The region outlined in black corresponds to the entire sequence encoded by the alternate exons, including the voltage sensor in the first domain. *B*, the conserved splicing motif in the first domain of sodium channels. Introns (thin black lines) and exons (squares) are to scale. In all cases the N exon precedes the A exon and they are separated by a short intron. Similar architecture is present in *SCN3A*, *SCN5A* and *SCN8A*, which also arose from duplications during vertebrate evolution. Numbers correspond to the current annotation of exons in Ensembl and NCBI. *C*, the identical sequences encoded by the A exons in human *SCN1A* (1A, top), *SCN2A* (2A, middle) and *SCN9A* (9A, bottom). The aspartate (D) which is consistently changed to a neutral amino acid (serine, S, or asparagine, N) is indicated by the box. *D*, the sequences encoded by the N exons in *SCN2A* (2A, top) and *SCN9A* (9A, bottom). In these two genes the sequences encoded by the N exons are identical in all 23 mammalian genomes surveyed. Only silent changes are observed, with 3 silent changes seen for all 23 copies of SCN2A and 2 changes seen in SCN9A (see methods). *E* and *F*, the sequences encoded by the N exon in *SCN1A* (NaV1.1) for 23 mammalian species where this exon could be unambiguously identified. With full length sequences aligned (*E*), and truncated exons not aligned (*F*). The changes in *E* are highlighted according to functional impact: conservative (green), not conservative (yellow), loss of an S4 arginine (purple). In some cases (Oc, Og, Cp, Cg, Mm, Rn) the exon could not be identified using standard megaBLAST parameters and the location of the N exon was ascertained using its proximity to the A exon in *SCN1A* (as described in *B*, and Fig. 1). It was not possible to identify the N or A exons in *SCN1A* from *Sus scrofula*. Species: Cj, *Callithrix jacchus*; Hs, *Homo sapiens*; Mml, *Macaca mulatta*; Nl, *Nomascus leucogenys*; Og, *Otolemur garnettii*; Pp, *Pan paniscus*; Pt, *Pan troglodytes*; Pan, *Papio anubis*; Pab, *Pongo abelii*; Sb, *Saimiri boliviensis*; Gg, *Gorilla gorilla;* Cp, *Cavia porcellus*; Cg, *Cricetulus griseus*; Mms, *Mus musculus*; Rn, *Rattus norvegicus*; Am, *Ailuropoda melanoleuca*; Bt, *Bos taurus*; Cl, *Canis lupus*; Ec, *Equus caballus*; Fc, *Felis cattus*; Oc, *Oryctolagus cuniculus*; Oa, *Ovis aries*; La, *Loxodonta Africana*. [Color figure can be viewed at wileyonlinelibrary.com]

First we asked whether the loss of the N exon from mouse and human orthologs of *SCN1A* is a coincidence, or if loss of this particular exon is more widespread. In humans the expression of this exon is reduced by the common rs3812718 polymorphism (Tate *et al*. 2005; Heinzen *et al*. 2007; Kasperaviciute *et al*. 2013), which disrupts the 5’ splice site consensus sequence (Zhang, 1998). In mice this exon contains frame shifts and stop codons (Gazina *et al*. 2015), which we confirmed were also present in the rat genome. Using the cluster of sodium channel genes in human chromosome 2 (Fig. 1), we identified the N exon in SCN1A in 23 mammalian genomes (Fig. 2). The N exon in *SCN1A* had frameshifts and stop codons in seven species (Fig. 2). The first voltage‐sensing arginine was removed from the S4 segment in a further six species, a substitution likely to be damaging for channel function (Gosselin‐Badaroudine *et al*. 2012). Only 10 of the 23 species did not contain amino acid substitutions that are likely to be deleterious in the N exon (Fig. 2). Further evidence for selection to reduce the N exon in *SCN1A* comes from the finding that a splice site polymorphism homologous with the human SNP rs3812718 has arisen independently in giant panda (Ailuropoda melanoleuca).

Does variability in the N exon of *SCN1A* reflect neutral selection that tolerates changes in the N exons of all neuronal sodium channels (making these N exons poorly conserved in general), or is the genetic damage specific to *SCN1A*? To answer this, we identified the corresponding N exons in *SCN2A* (6N) and *SCN9A* (5N) in the same 23 species and asked whether a similar degree of variation occurred as in *SCN1A*. No species had any amino acid substitutions in either *SCN2A* or *SCN9A*, indicating that, unlike *SCN1A*, the N exons in these genes may be subject to selection to *remove* variation (Fig. 2). Compared to *SCN2A* and *SCN9A*, the number of species with potentially damaging changes in the N variant of *SCN1A* was significantly larger (*P* < 0.0001; Fisher's exact test, correction for multiple tests: *P* < 0.0002, counting rodents as a single ancestral loss).

To estimate whether the changes in the N exon of *SCN1A* could be considered indicators of selective pressure, or were simply an outcome of genetic drift, we used the pooled data from all of the species where the exon could be aligned to compare the rate of non‐synonymous (Ka) and synonymous (Ks) changes in this exon (see methods). The Ka/Ks ratio is a proxy for detecting genes under directional selection, with a Ka/Ks ratio greater than 1 considered indicative that a gene may be under strong selection. This comparison yields a Ka/Ks = 2.4424 for the N exon of *SCN1A*, and indicates that this exon is under strong selective pressure in mammals (Liberles & Wayne, 2002). In contrast, neither *SCN2A* nor *SCN9A* contain non‐synonymous changes in their N exons and consequently their Ka/Ks ratio is 0, indicating purifying selection may be *stabilizing* the N exons in these genes. Thus, the N exon in *SCN1A* appears to be alone in being under strong selection for removal among mammalian genomes. This may mean the splicing has a functionally different consequence in *SCN1A* that is deleterious, or that a conserved functional change imposed by splicing has a distinct effect in *SCN1A* because of the role Nav1.1 plays in the nervous system.

### Splicing has little effect on macroscopic currents and voltage dependence

**Figure 3:**
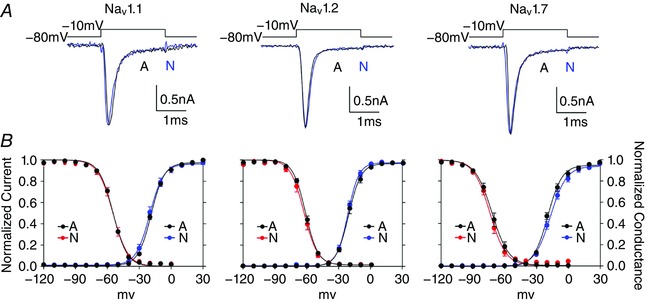
Splicing does not alter macroscopic kinetics or voltage dependence *A*, representative traces from HEK cells expressing A (black) and N (blue) variants of Nav1.1 (left), Nav1.2 (middle) and Nav1.7 (right) evoked by a 2 ms step from −80 mV to −10 mV. Macroscopic properties of the currents were largely indistinguishable for individual traces. Currents are from cells selected to have similar peak currents for comparison, but are not scaled. *B*, voltage dependence of steady state inactivation (N = red and A = black) and activation (N = blue and A = black) for splice variants are similar and not robustly affected by splicing in any of the three channels in these conditions (Table 1). [Color figure can be viewed at wileyonlinelibrary.com]

To ask whether the effects of splicing were similar in different sodium channels, we compared N and A splice variants of the three different sodium channels in similar conditions. We used individual channels in non‐neuronal cells to isolate the effects of splicing on intrinsic properties of pure populations of the pore forming subunits of channels. As reported previously, switching exons N and A does not have pronounced effects on macroscopic currents or on the voltage dependence of Nav1.1 or Nav1.7 (Chatelier *et al*. 2008; Fletcher *et al*. 2011) (Fig. 3). Nav1.2 is a potential outlier, because splicing can modify the voltage‐dependence of inactivation (Xu *et al*. 2007), but in our recording conditions the voltage‐dependence of variants was similar (Fig. 3).

Sodium currents recorded at physiological temperatures represent challenges for voltage clamp recordings, and some errors due to series resistance are inevitable. For this reason, as well as keeping series resistance low (typically 1–2 MΩ, often below 1 MΩ) and currents small (typically 1–2 nA), we employed three additional strategies to control for errors introduced by series resistance, and importantly, to ensure series resistance did not introduce artificial differences between splice variants: firstly, we carried out all recordings while blinded to variants transfected to reduce bias; secondly, we repeated all recordings with three different sodium channels, and while the different channels clearly have different voltage dependencies (as is expected) the splice variants from each individual channel did not differ, and thirdly, our plots of the voltage dependencies of activation, which are particularly sensitive to failure of control of voltage during steps with excessive series resistance, do not show larger variation in the regions near the V_½_ max where errors due to failure to successfully clamp cells increase variability (Fig. 3 *B*). For these reasons, although as for any voltage clamp recording we cannot claim to have identified the absolute values of the voltage dependencies of the channels, our data are strongly supportive of the relative similarities of splice variants, and confirm the differences between Nav1.1, 1.2 and 1.7 (Table 1).

**Table 1:** Voltage dependence of activation and steady state inactivation for splice variants are similar

	Nav1.1				Nav1.2				Nav1.7			
	A	SEM	N	SEM	A	SEM	N	SEM	A	SEM	N	SEM
*V*_½,act_ (mV)	−16.5	1.4	−17.9	1.9	−21.5	3.7	−21.3	1.4	−15.9	2.5	−16.2	3
*V*_½,inact_ (mV)	−55.4	1.1	−55.1	2.4	−61.2	1.6	−62.9	2.4	−71.8	3.6	−74.3	3.9
*n*	6		6		9		7		6		7	

### Effects of splicing on recovery after long inactivating pre‐pulses are not conserved

**Figure 4:**
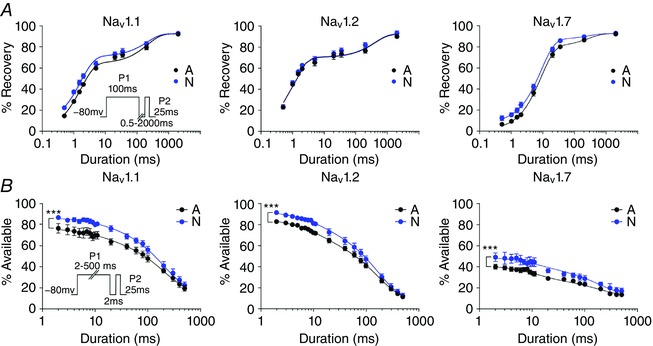
Splicing has a conserved effect on channel availability after inactivation *A*, for both Nav1.1 (left) and 1.7 (right), N variants showed more recovery than A variants after short intervals. Variants of Nav1.2 (middle) were not different. Values for all fits are in Table 3. Nav1.1 A *n* = 10, N *n* = 9; Nav1.2 A *n* = 7, N *n* = 6; Nav1.7 A *n* = 5, N *n* = 8. *B*, the N variants of all three channels showed significantly more availability after shorter pre‐pulses (^***^two‐way ANOVA, *P* < 0.001). The curves shown were fitted with single exponentials with the offset and rates fixed (all rates from free fits are given in Table 2). Only the amplitude of the exponential was significantly different for the splice variants (i.e. the proportion of channels inactivating with this fast time course, as opposed to slow inactivated states, for example). Nav1.1: A, *n* = 6, N, *n* = 6; Nav1.2: A, *n* = 10, N, *n* = 9; Nav1.7: A, *n* = 7, N, *n* = 10. [Color figure can be viewed at wileyonlinelibrary.com]

The N variant of Nav1.1 recovers from 100 ms inactivating pre‐pulses more rapidly than the A variant (Fletcher *et al*. 2011). We confirmed this, and asked whether similar effects are imposed by splicing in Nav1.2 and Nav1.7 (Fig. 4). While Nav1.7 did recover more slowly from inactivating pre‐pulses overall, the difference between N and A splice variants was conserved for this channel, and was similar to that observed in Nav1.1 where the earliest phases of fast recovery from inactivation were more pronounced for the N variant than for the A variants. For both Nav1.1 and Nav1.7 the difference in recovery was most evident after brief recoveries, and was obscured after longer recovery intervals (>100 ms). However, unlike Nav1.1 and Nav1.7, splice variants of Nav1.2 did not reproduce this difference in recovery (Fig. 4). The N and A variants of Nav1.2 showed similar amounts of recovery at all time intervals. This indicates that recovery from inactivation is not a conserved impact of alternative splicing, but does not highlight an aspect of splicing that could explain the selectively deleterious nature of exon N in Nav1.1, as the change seen for Nav1.1 was also present in Nav1.7.

### Splicing has conserved effects on availability after short depolarizations

In neurons the most physiologically relevant depolarizations are much shorter than 100 ms. More typical action and synaptic potentials last on the order of a few milliseconds. We therefore asked if shorter depolarizations, consistent with brief synaptic currents or action potentials (APs), are able to provoke different responses from splice variants of Nav1 channels. An additional reason for focusing on shorter inactivating prepusles was the observation that for both Nav1.1 and Nav1.7 where splicing did have an effect on recovery, this effect was selective for the fast component (Table 2), suggesting splicing may selectively alter fast inactivation, which is rapid both in onset and recovery.

**Table 2:** Parameters of single exponential fits describing channel availability after 2 ms recovery from pre‐pulses of different durations

	Nav1.1‐N		Nav1.1‐A		Nav1.2‐N		Nav1.2‐A		Nav1.7‐N		Nav1.7‐A	
	Value	SEM	Value	SEM	Value	SEM	Value	SEM	Value	SEM	Value	SEM
**Y_0_**	0.22	0.02	0.19	0.02	0.13	0.02	0.13	0.01	0.18	0.01	0.14	0.01
**A**	0.640	0.022	0.547	0.018	0.764	0.012	0.689	0.014	0.323	0.012	0.243	0.010
**τ (ms)**	155	13	164	15	111	10	106	8	98	10	92	9
${\bar{R}}^{2}$	*0.991*		*0.989*		*0.992*		*0.993*		*0.977*		*0.972*	

Fits were carried out with all parameters allowed to vary. To better compare the goodness of fits with the different degrees of freedom the adjusted *R*‐square statistic (${\bar{R}}^{2}$) is given. In this instance only the amplitude (A) differed significantly between the two variants. The confidence intervals for the other parameters (Y_0_ and τ) overlapped.

**Table 3:** Parameters of bi‐exponential fits to recovery from long (100 ms) inactivating pre‐pulses

	Nav1.1‐N		Nav1.1‐A		Nav1.2‐N		Nav1.2‐A		Nav1.7‐N		Nav1.7‐A	
	Value	SEM	Value	SEM	Value	SEM	Value	SEM	Value	SEM	Value	SEM
**Y_0_**	0.044	0.040	0.003	0.036	0	0.081	0	0.069	0.079	0.007	0.012	0.009
**A_F_**	0.65	0.04	0.67	0.36	0.73	0.08	0.71	0.06	0.78	0.01	0.79	0.02
**τ_F_ (ms)**	1.5	0.2	1.9	0.2	1.1	0.2	1.0	0.2	8.7	0.4	8.9	0.6
**A_S_**	0.23	0.03	0.25	0.03	0.20	0.04	0.20	0.04	0.07	0.01	0.12	0.02
**τ_S_ (ms)**	218	69	272	95	315	202	541	559	273	139	265	100
${\bar{R}}^{2}$	*0.994*		*0.994*		*0.978*		*0.981*		*0.999*		*0.999*	

${\bar{R}}^{2}$ is the adjusted R‐square statistic used to improve comparison of fits with different degrees of freedom. The requirement for two exponentials to adequately fit recovery from inactivation is consistent with a fast and a slow component of recovery from inactivation, with the slow component poorly defined in our conditions.

Indeed, testing recovery after a fixed 2 ms interval after pre‐pulse durations between 2 and 50 ms, revealed marked differences between splice variants of all three channels, with the N variants consistently more available than the A variants (Fig. 4). In contrast, longer pre‐pulses (>100 ms) obscured the differences between the variants of all three channels, further supporting a specific role for alternative splicing in modifying fast inactivated states. The difference in availability after short pre‐pulses was similar (∼10 %) across all three channels, in spite of Nav1.7 showing overall less availability than Nav1.1 and Nav1.2. Taken together, these data suggest the difference between splice variants is due to more rapid availability of the N variants after fast inactivation, a difference which can be obscured as longer depolarizations shift the channels into slow inactivated states. The conserved nature of the difference imposed by splicing, further supports the possibility that any deleterious effect of the N exon in *SCN1A* is not due to a qualitatively different consequence of that exon on Nav1.1 channels *per se*, but more likely due a deleterious impact arising from the specialized role Nav1.1 channels play in the nervous system.

### The N variant of all three channels supports more activity during rapid trains of depolarizations

**Figure 5:**
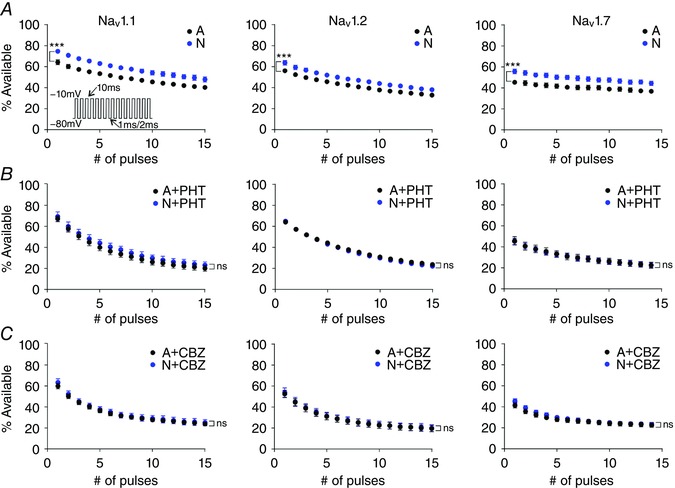
Splicing changes response to fast trains of stimuli and has consistent effects on pharmacology of all three channels *A*, N variants of all three channels are able to support larger currents during rapid trains of depolarizing steps. The first step in all cases was normalized to 100%, with responses to the following steps shown scaled. The difference between variants was consistent for all three channels and persisted for the duration of the train (^***^two‐way ANOVA, *P* < 0.0001). Nav1.1: A, *n* = 8, N, *n* = 8; Nav1.2: A, *n* = 11, N, *n* = 9; Nav1.7: A, *n* = 6, N, *n* = 8. The recovery intervals used were 1 ms for Nav1.1 and Nav1.2, but 2 ms was allowed for Nav1.7 channels, which show little current after a 1 ms recovery. *B* and *C*, addition of either phenytoin (*B*) or carbamazepine (*C*) to Nav1.1, Nav1.2, or Nav1.7 reduces channel availability after the first step, and obscures the difference between splice variants during the train (ns, *P* > 0.05, two‐way ANOVA). Each drug was applied at 30 μm, a concentration which approximates half‐maximal inhibition (Thompson *et al*. 2011). [Color figure can be viewed at wileyonlinelibrary.com]

The different availability after single short depolarizations raises the possibility that splicing can also change the ability of sodium channels to open repeatedly during trains of stimuli, such as during bursts of APs. To investigate this possibility, we delivered trains of brief depolarizing steps with short recovery intervals and compared the availability of the splice variants (Fig. 5). Because Nav1.7 did not recover sufficiently in with the shortest intervals of 1 ms, variants of this channel were recorded with longer recovery intervals of 2 ms. Indeed, although our holding potential of −80 mV is in the range of physiological potentials where neurons may rest, this potential is within a range where a significant portion of Nav1.7 will be in slow inactivated states (Hampl *et al*. 2016). However, for all three channels, the difference in channel availability was evident after a single step and was sustained for the entire train, with the A variants remaining less available than the N variants (*P* < 0.01 for all three channels, two‐way ANOVA). These results thus imply that splicing may have a conserved role in the ability to sustain rapid firing across all three channels. However, given the different amounts of slow inactivation for the three channels, in particular for Nav1.7 which has relatively more slow inactivation, the overall rate of activity supported by the channels may be different, with either variant of Nav1.7 channels being unlikely to support firing at frequencies as rapid as variants of Nav1.1 or 1.2. Thus while the N variants support approximately 10% more availability for all three channels, this 10% increase is imposed upon a different background availability determined by the identity of the pore forming subunits.

### For all three channels the difference between variants is occluded by anti‐epileptic drugs

The N and A variants of Nav1.1 have different sensitivities to anti‐epileptic drugs (Thompson *et al*. 2011). These drugs bind to and stabilize the inactivated states of Nav channels (Kuo & Bean, 1994). If the difference in splicing is mediated through a conserved impact of splicing on the stability of inactivation, any altered drug response should also be conserved among variants of all three sodium channels. We tested this hypothesis, using the same trains of short depolarizations to compare the use‐dependent block by phenytoin and carbamazepine. For all channels the presence of either drug, at concentrations within the therapeutic range (Thompson *et al*. 2011), occluded the difference between the splice variants (Fig. 5). This is consistent with the splicing having an effect on stability of the inactivated states which is occluded by the presence of the drugs that further stabilize inactivated states.

### Modelling suggests splicing in the first domain could change the rate of unbinding of the inactivation domain

**Figure 6:**
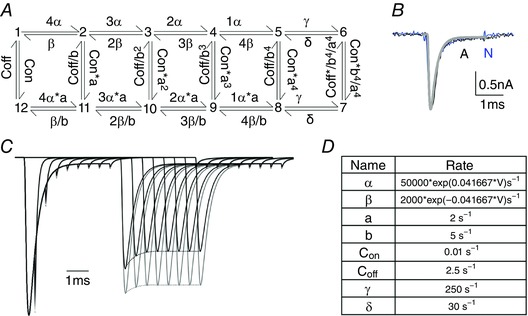
A model of sodium channel gating indicates that modification of a single gating step may be sufficient to modify channel availability without altering other parameters *A*, the scheme, as adapted from Carter *et al*. (2012) and adjusted for our human Nav1.1 channels recorded in HEK cells at 37°C. The top left position (1) corresponds to resting, closed channels with all four voltage sensors down (de‐activated) and the inactivation domain off (i.e. channels are not inactivated, or are de‐inactivated). The only state which passes current is state 6 (top right), which channels briefly visit upon depolarization. The bottom row of states (7–12) all have the inactivation domain bound (‘on’). The two splice variants of Nav1.1 were modelled by changing the rate at which channels move between states 1 and 12 (i.e. the rate at which the inactivation domain binds to or is released from the inner pore of channels with all four voltage sensors in the resting, ‘down’, configuration). The transitions for A variants are 0.35 times the rates for transitions between these states for the N variants (exact values: A(1–12) = 0.01 × 350 s^−1^; N(1–12) = 0.01 × 1000 s^−1^; A(12–1) = 2.5 × 350 s^−1^; N(12–1) = 2.5 × 1000 s^−1^). *B*, the model accurately predicts macroscopic currents produced by either splice variant of Nav1.1. A = thin black line, N = thin blue line, model = thick grey line overlay. Macroscopic currents predicted by the model for both variants during a single step were identical. *C*, changing the rate of transitions between states 12 and 1 is able to replicate the increased availability of the N variant relative to the A variant after short inactivating pre‐pulses of variable duration. Note that the predicted currents in response to the initial step are identical (the blue traces exactly overlay the black traces during the response to the first step). *D*, the full rates used for the model for the N variant of Nav1.1. Rates for the adult variant were identical except for the transitions between states 12 and 1. Note the units are in seconds for these rates. [Color figure can be viewed at wileyonlinelibrary.com]

How can splicing change the availability of channels after short depolarizations without altering the voltage‐dependence or macroscopic kinetics of the currents? We used a model of Nav1 channels (Carter *et al*. 2012) to ask whether it is possible, in principle, to reproduce this change in availability without altering other kinetic parameters. For simplicity, because the effects of splicing appear confined to fast inactivated states, and are obscured by slow inactivated states resulting from longer depolarizations, we chose a model which includes only active and fast inactivated states (Fig. 6). We adjusted the rate constants to match the predicted currents from the model with Nav1.1 currents recorded in HEK cells (Fig. 6).

**Figure 7:**
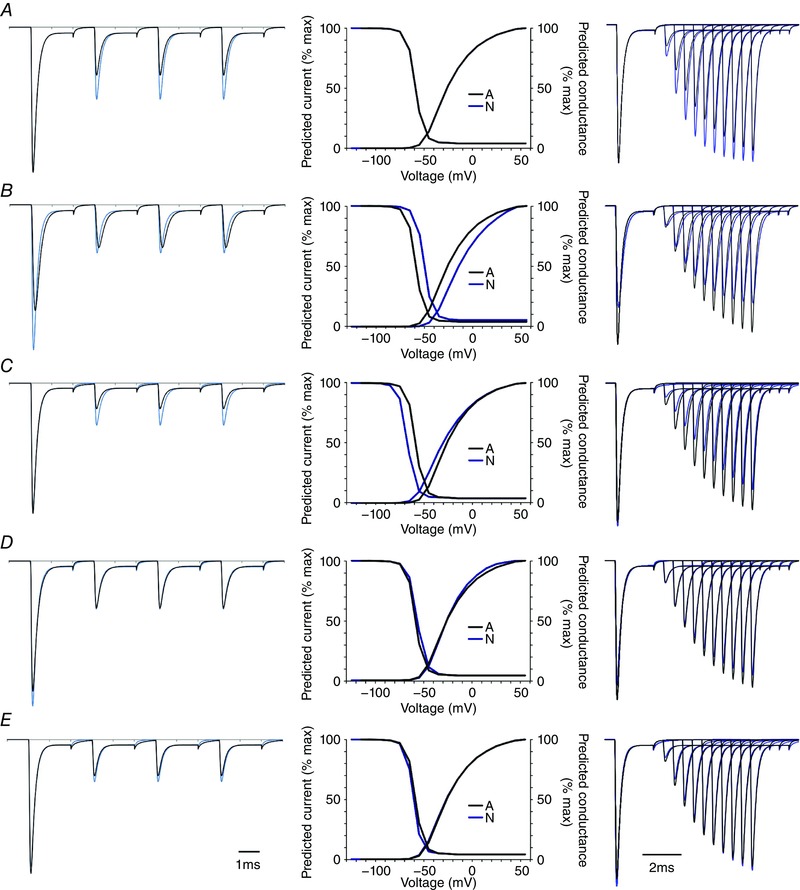
Changing transitions between states 12 and 1 reproduces differences seen with splicing, while changing most other rates affect multiple parameters *A*, changing the transitions between states 12 and 1, as described in Fig. 6, alters availability during a train of brief depolarizations (left) but does not change voltage dependence of activation and inactivation or the macroscopic kinetics of currents (middle). The predicted voltage dependence of activation and steady state inactivation were identical for both variants. Both black (A) and blue (N) lines are shown, but overlay exactly. This parameter is also sufficient to reproduce the altered change in rate of recovery from fast inactivation (right). *B*, changing the rate of S4 movement in response to depolarization (α) by 50% does change the availability of channels during fast trains of pulses, but this parameter also changes rate of activation during a voltage step, and voltage dependence of both steady state inactivation and activation. The effect on recovery from inactivation is minimal. *C*, altering the voltage dependence of downward movements of S4 segments (β) has marked effects on availability during fast trains of steps (left), and recovery from inactivation (right), but also changes the voltage dependence of steady state inactivation (middle). *D*, changing the rate of pore opening (γ) changes the total availability of channels (note initial peak is different, left panel, arrow), but has relatively modest effects on availability during trains of activity (left), and scaling the initial peaks removes the difference in recovery from inactivation (right). *E*, altering the rate of pore closing (δ) has very small effects on availability during trains (left), as well as small effects on both voltage dependence of inactivation (middle) and recovery (right). The effects of changing δ are likely to be small because most channels inactivate before closing. Currents predicted with the A values are in black throughout. The horizontal scale is the same throughout, and vertical scale represents proportion of simulated channels activated. [Color figure can be viewed at wileyonlinelibrary.com]

**Figure 8:**
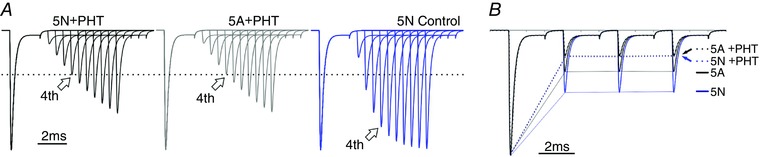
Modelling effects of anti‐epileptic drugs can obscure the differences predicted by splicing *A*, the predicted reduction in recovery as the transitions between states 1 and 12 are further slowed by a factor of 0.01. Note that in the presence of the modelled ‘PHT’ the response to predicted 4th step is identical for rates for both the N and A variants. The response predicted for the N variant in absence of the ‘PHT’ effect is shown for comparison, where the 4th interval has recovered to a much greater extent. *B*, tThe slowing of the modelled ‘PHT’ effect eradicates the difference between the two splice variants predicted by the model. The initial predicted responses are as in Fig. 8, but the further reduction in availability is overlaid. Both the N + PHT and A + PHT overlay exactly in this panel. [Color figure can be viewed at wileyonlinelibrary.com]

As expected, changes made to most of the transition rates altered both the voltage‐dependence and macroscopic kinetics of the channels (Fig. 7). For example, altering either α or β, or the voltage dependence of up and down movements of each S4 segment, changes macroscopic kinetics (α) and voltage dependence of activation (α, and to a lesser extent, β) and steady state inactivation (both α and β, Fig. 7). Changing γ or δ (pore opening or closing), could change maximal currents (γ), but had little effect on stability of inactivation (γ and δ, Fig. 8).

However, the rate limiting step determining channel availability after inactivation is the return of the fully *de*activated channel from the *in*activated state (i.e. state 12 in Fig. 6), back to the state which is fully deactivated and non‐inactivated (i.e. state 1 in Fig. 6). We found that reducing the rate of transitions between states 1 and 12 by a factor of 0.35 was sufficient to change channel availability after short inactivating steps but had little or no impact on other parameters (Figs 6 and 7). This change also reproduced the sustained difference in availability during trains of short stimuli in a manner qualitatively similar to that seen for splice variants in HEK cells (Fig. 7).

Finally, we asked whether we could model the effects of anti‐epileptic drugs. We did this simply by further slowing the transitions between states 12 and 1, to mimic the reduced recovery from inactivated states. We imposed a further slowing of 0.01 times the rates for each splice variant, but preserved the 0.35‐fold difference between the two variants. In these conditions, the model predicted that the overall slowing of the anti‐epileptic drugs could obscure the difference between the variants produced by splicing, and the model predicted channel availability for the variants to be similar both in recovering from steps, and in trains of fast steps (Fig. 8).

Thus, our data reveal a general rule for the function of alternative splicing in sodium channel genes, and an evolutionary caveat. In three separate channels, we have shown that inclusion of the N exon increases channel availability during high frequency stimulation. The conserved nature of this change in three different channels suggests the selective pressure against the N exon in *SCN1A* is due to indirect consequences of increasing the availability of Nav1.1 channels.

## Discussion

While splicing in the first domain of sodium channels is generally extremely well conserved, we report that in mammalian genomes, *SCN1A* appears to be under selective pressure in diverse species to remove the alternate N exon. Despite this molecular evidence for a maladaptive role of the N exon in *SCN1A*, alternative splicing has a conserved functional effect across human Nav1.1, Nav1.2 and Nav1.7 channels. Channels containing the N exon are more available during high frequency trains of stimuli, and the differences between variants are occluded by two anti‐epileptic drugs that interfere with recovery from inactivation. Our modelling suggests that splicing in the D1S3‐S4 linker modifies the rate at which channels leave the inactivated states.

At present it is not known why the N exon of SCN1A is being lost in mammals. While sequencing studies do support a rapid increase in the disruptive allele of rs3812718 in human populations (the ancestral G allele only remains in ∼50% of chromosomes), clinical genetics do not suggest a strong negative consequence from inclusion of the N exon in humans. In fact despite the widespread inclusion of the A allele in human chromosomes, a weak association has been reported suggesting the G allele has a protective effect, conferring reduced risk of epilepsy (particularly epilepsy with febrile seizures) in two meta‐analysis studies (Baum *et al*. 2014; Tang *et al*. 2014). Our results suggest expression of the N exon in Nav1.1 may allow more rapid firing of neurons in the face of intense synaptic excitation, which given the predominant location of this channel in interneurons (Yu *et al*. 2006; Ogiwara *et al*. 2007), could help protect against seizure activity, but even if this is the case, this protection does not appear sufficient to overcome the evolutionary pressure to remove the N exon from mammalian *SCN1A*.

Effectively, inclusion of the N exon confers a small gain of function effect on channels by increasing their availability. For *SCN2A*, and *SCN9A* (and potentially also *SCN3A* and *SCN8A* where splicing is conserved), this increase in function appears well‐tolerated, and even appears strongly conserved in mammalian evolution. In contrast, in *SCN1A*, the change appears detrimental, and because the intrinsic changes to the channel are similar for all three genes, it is likely this is due instead to constraints on the role of *SCN1A*. Indeed this gene appears highly intolerant to changes (Lek *et al*. 2016). Loss of function in *SCN1A* is strongly associated with epilepsy, but gain of function mutations are also known to be deleterious, as rare mutations with gain of function effects in Nav1.1 are associated with familial migraine (Cestèle *et al*. 2013). This raises the testable hypothesis that the G allele of rs3812718 may be associated with altered risk of migraine or with different response of migraine to treatment with sodium channel blockers. Our data suggest that revisiting rs3812718 for association with diseases other than epilepsy may be informative.

Splicing at this site is associated with inherited neurological diseases in the other channels as well. Splicing in *SCN2A* modifies the time course of infantile epilepsy (Xu *et al*. 2007), while splicing in *SCN9A* can change the age of onset of intractable pain (Choi *et al*. 2010). In mice, removal of the N exon in *SCN2A* in mice leads to a change in seizure threshold (Gazina *et al*. 2015), and the effects of permanent changes in splicing on neuronal circuits *in vivo* highlight the challenges of translating pure biophysical effects in cells to specific consequences in the neuronal milieu where splice variants may interact differently with kinases, g‐proteins or other modulating pathways. This splicing is co‐regulated in multiple channels both during development (Gazina *et al*. 2010) and disease (Gastaldi *et al*. 1997; Tate *et al*. 2005). In the *SCN5A* cardiac channels, dysregulated splicing is associated with cardiac arrhythmia (Freyermuth *et al*. 2016). The preservation of this splicing in these genes, despite their divergence to different functional roles in distinct cellular populations, strongly suggests that the functional role of this splicing supersedes the specialization of each channel. Although splicing in these channels has been studied in isolation (Xu *et al*. 2007; Chatelier *et al*. 2008; Jarecki *et al*. 2009; Choi *et al*. 2010; Fletcher *et al*. 2011), no general rule for its functional consequences has been proposed. One reason for the lack of consensus may be because previous studies of NaV1.1 and NaV1.7 splice variants at room temperature have shown conflicting results, whereas we show a robust increase in availability in the N variant of all three isoforms at physiological temperatures. This highlights the importance of performing experiments at elevated temperatures as not all data may be extrapolated from room temperature recordings.

*SCN1A* may not be the only channel for which more rapid recovery from inactivation produced by the aspartate to asparagine switch is deleterious. *SCN4A*, which encodes Nav1.4 the channel that predominates in skeletal muscle, has only transcripts encoding aspartate (equivalent of exon A) at this site reported in databases. The N exon appears to have been completely lost in these channels. Finally, the TTX‐resistant channels encoded by *SCN10A* and *SCN11A* also do not have alternative splicing at this site; however these channels have much slower macroscopic kinetics of inactivation, and consequently may not be sensitive to changes that alter fast inactivated states.

At the molecular level, the modelling suggests functional specialization of the first the domain of these channels. Studies of gating currents have suggested that the voltage sensor in the fourth domain (D4S4) is most important for limiting the rate of onset and recovery from inactivation (Capes *et al*. 2013). We can now add to what is known about the specialization of the different domains of these channels by proposing that the first domain (D1) could be important for *release* from inactivation. A possible mechanistic explanation is that, while the S4 in the fourth domain could be the last to activate and thus initiates binding of the inactivation domain (Chanda & Bezanilla, 2002; Capes *et al*. 2013), it is possible that unbinding from D1 may be the rate‐limiting step for dissociation of the inactivation domain from the inner side of the deactivated channel. A related, but slightly less well conserved, splicing event in the first domain of the cardiac sodium channel (SCN5A/Nav1.5) also affects the D1S3‐S4 linker. This splicing, which unlike splicing in the neuronal channels incorporates a positively‐charged lysine in the N exon, has several effects on channel kinetics and voltage dependence, as well as *slowing* recovery from inactivation (Onkal *et al*. 2008). Thus, in spite of the divergence in the sequence of the N exon in cardiac channels, the role of D1 in stability of inactivation appears conserved (albeit combined with additional functional effects of splicing not seen for neuronal channels in our conditions). Finally, the drive for evolution to exploit the specialized roles of the domains of sodium channels to control inactivation is supported by a paralogous splicing event in Drosophila changes the S3‐S4 linker in the third domain of the sodium channel (Copley, 2004). Recent work has shown this splicing changes amount of persistent sodium current produced, and has pronounced effects on neuronal excitability (Lin *et al*. 2012). Thus splicing in D3 suggests this domain may regulate the total *amount* of inactivation, rather than its rate of onset or release.

In spite of the conserved effects on channel availability, we cannot rule out other roles for this splicing in contributing to distinct functions in different neuronal populations, or in regulating interactions with other proteins. For example, in Nav1.7, splicing has been shown to modify phosphorylation (Chatelier *et al*. 2008) and interactions with β subunits (Farmer *et al*. 2012).

Our data suggest there is a general rule that alternative splicing in the DIS3‐S4 linker of neuronal sodium channels modifies their availability after short depolarizations, implicate the first domain in determining channel availability, and predict that similar effects will be found in *SCN3A* and *SCN8A* for this splice site.

## Additional information

### Competing interests

The authors declare no competing financial interests.

### Author contributions

A.L., G.L. and S.S. conceived and designed the work; A.L and G.L. performed experiments; A.L., G.L. and S.S. analysed experimental data; A.L., G.L., and S.S. drafted and wrote the paper. A.L., G.L. and S.S. have approved the final version of the manuscript, agree to be accountable for all aspects of the work, and all qualify for authorship.

### Funding

This work was supported by fellowships from the Worshipful Company of Pewterers and from the Royal Society (UF090203 to SS), an MRC‐funded PhD studentship (A.L.), as well as by grants from the MRC MR/L01095X/1, MR/L003457/1 and Wellcome Trust 104033/Z/14/Z. The authors declare no competing financial interests.

**Translational perspective**

Splicing in SCN1A leads to increased channel availability and may be detrimental in mammalian nervous systems. This is consistent with recent findings suggesting mutations linked to gain of function in this gene can cause migraine. Splicing may also affect migraine and its treatment. This is particularly relevant as many patients may have a common polymorphism that changes the ratio of splice variants in SCN1A, and which may affect the dosage of some drugs that have been used to treat migraine (e.g. carbamazepine). Our work raises the hypothesis that there may be a genetic association between SNPs in SCN1A that alter splicing and susceptibility to migraine, with genotypes favouring inclusion of the ‘N’ or ‘neonatal’ exon increasing risk. Splicing also interacts with pharmacology in a manner that suggests many sodium channels may be more affected by common anti‐epileptic drugs after seizures. Potential strategies for targeting splice variants rather than different types of channels may be valuable routes forward for developing more selective sodium channel drugs which could be more effective in tissue where the N exons are broadly upregulated.
